# Cell Penetration Properties of a Highly Efficient Mini Maurocalcine Peptide

**DOI:** 10.3390/ph6030320

**Published:** 2013-03-18

**Authors:** Céline Tisseyre, Eloi Bahembera, Lucie Dardevet, Jean-Marc Sabatier, Michel Ronjat, Michel De Waard

**Affiliations:** 1 Unité Inserm U836, Grenoble Institute of Neuroscience, Université Joseph Fourier, La Tronche, Chemin Fortuné Ferrini, Bâtiment Edmond Safra, 38042 Grenoble Cedex 09, France; 2 Labex Ion Channel Science and Therapeutics, Nice, France; 3 Université Joseph Fourier, Grenoble, France; 4 Inserm U1097, Parc scientifique et technologique de Luminy, 163, avenue de Luminy, 13288 Marseille cedex 09, France; 5 Smartox Biotechnology, Biopolis, 5 Avenue du Grand Sablon, 38700 La Tronche, France

**Keywords:** maurocalcine, hadrucalcin, toxin, cell penetrating peptide, F98 cells, glioma, analogs

## Abstract

Maurocalcine is a highly potent cell-penetrating peptide isolated from the Tunisian scorpion *Maurus palmatus*. Many cell-penetrating peptide analogues have been derived from the full-length maurocalcine by internal cysteine substitutions and sequence truncation. Herein we have further characterized the cell-penetrating properties of one such peptide, MCa_UF1-9_, whose sequence matches that of the hydrophobic face of maurocalcine. This peptide shows very favorable cell-penetration efficacy compared to Tat, penetratin or polyarginine. The peptide appears so specialized in cell penetration that it seems hard to improve by site directed mutagenesis. A comparative analysis of the efficacies of similar peptides isolated from other toxin members of the same family leads to the identification of hadrucalcin’s hydrophobic face as an even better CPP. Protonation of the histidine residue at position 6 renders the cell penetration of MCa_UF1-9_ pH-sensitive. Greater cell penetration at acidic pH suggests that MCa_UF1-9_ can be used to specifically target cancer cells *in vivo* where tumor masses grow in more acidic environments.

## 1. Introduction

**Figure 1:**
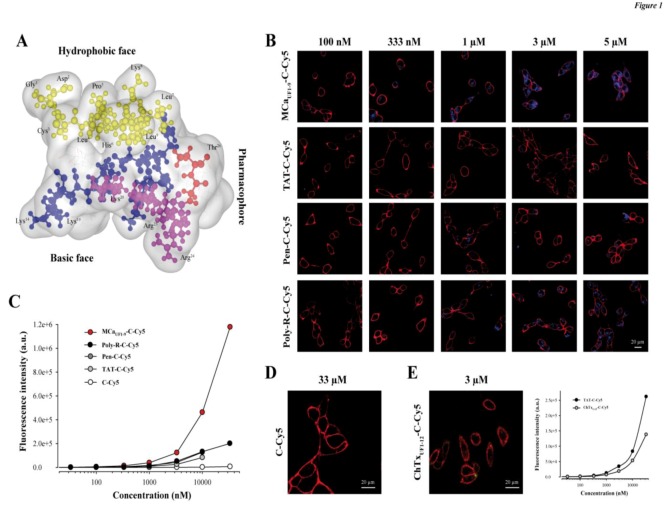
The hydrophobic domain of MCa is an efficient CPP. (**A**) Schematic representation of MCa 3D structure. The hydrophobic domain (from amino acid residue 1 to 9) is shown on top. Residues are in yellow. Shown also are the basic face (basic amino acid residues are in blue or in pink) and the pharmacophore (residues identified as interacting with the ryanodine receptor are in red or in pink). Pink residues belong both to the pharmacophore and the basic face. (**B**) Confocal microscopy images illustrating the penetration of four different peptides labeled with Cy5 at various concentrations into glioma F98 cells (blue color). Incubation times were 2 h for each peptide/concentration. Images were taken immediately after washout of the extracellular peptide. The plasma membrane is labeled with concanavalin-A-rhodamine (red color). (**C**) Dose-dependent penetration of each peptide-cargo complex in F98 cells as assessed by flow cytometry. A control Cys-Cy5 is also provided. (**D**) Absence of cell penetration of 33 µM Cys-Cy5 (a single Cys residue linked to Cy5—abbreviated C-Cy5) evaluated by confocal microscopy. (**E**) Lack of cell penetration of 3 µM ChTx_UF1-12_-C-Cy5 as determined by confocal microscopy (right panel). Quantitative analysis of F98 ChTx_UF1-12_-C-Cy5 fluorescence as determined by flow cytometry. Internalization of the ChTx_UF1-12_-C-Cy5 peptide is lower than TAT-C-Cy5 but not negligible.

Maurocalcine (MCa) is a 33 amino acid residue peptide that was isolated in 2000 from the venom of the Tunisian chactid scorpion *Scorpio maurus palmatus* [1]. It folds according to an ‘Inhibitor Cystine Knot’ (ICK) motif [2] and contains three disulfide bridges connected by the following pattern: C_1_-C_4_, C_2_-C_5_ and C_3_-C_6_ [3]. Based on high amino acid sequence and pharmacological target similarities, MCa belongs to a larger family of scorpion toxins that also includes imperatoxin A (from *Pandinus imperator*) [4], opicalcine 1 and opicalcine 2 (from *Opistophthalmus carinatus*) [5], hemicalcin [6] and hadrucalcin [7]. All these peptides act on ryanodine receptors resulting in pharmacological activation. These receptors are calcium channels located in the membrane of the endoplasmic reticulum. They control Ca^2+^ release from internal stores and therefore a large number of cell functions [7,8,9,10]. Binding of MCa on the ryanodine receptor type 1 occurs on protein cytoplasmic domains. Because MCa acts within seconds, once applied to the extracellular medium, it was soon obvious that it had to cross the plasma membrane very efficiently in order to activate the ryanodine receptor [11]. An additional curiosity of MCa lies into the fact that there is an intriguing sequence homology with a domain of the L-type voltage-gated calcium channel from the skeletal muscle (domain A). This channel lies in the plasma membrane, while domain A is found underneath the membrane in the cytoplasm within a loop that has been recognized as extremely important for the process of excitation-contraction coupling [12,13]. While the role of domain A in excitation-contraction coupling is still unclear, it is however surprising and stimulating to observe that peptides containing domain A sequence act with quite a lot of similarities to MCa on the ryanodine receptor type 1 [14,15]. According to the ^1^H-NMR solution structure, MCa is rigidly structured by the three disulfide bridges and contains three β-strands, comprising the following stretches of amino acid residues: 9–11 (strand 1), 20–23 (strand 2), and 30–33 (strand 3). MCa has an incredibly stable structure since it cannot be denatured, even at high temperatures up to 100 °C or extreme pH values [16]. Interestingly, the peptide is highly enriched in basic amino acid residues, more than a third of the amino acids being either lysine (seven out of 33) or arginine residues (four out of 33). The histidine residue at position 6 is susceptible, depending of the environmental pH, to introduce an additional positive charge to the peptide by protonation. Interestingly, stretches of positively charged residues seem to confound with the three MCa β-strands. The 3D structure of MCa also strikingly highlights the asymmetrical distribution of the positive charges at its surface: one face is highly basic, while the opposite face is rather hydrophobic. If, in addition, one depicts the amino acid residues important for the ryanodine receptor activation [17], then it appears that the peptide can be schematically represented with three domains: one hydrophobic head that tops the peptide, a larger second face, mainly basic, and a third side domain that contains the pharmacophore (Figure 1A).

Besides its Ca^2+^ channel activity, MCa is also used as a cell-penetrating peptide (CPP). Its properties have been investigated in detail. It was soon discovered that MCa could act as vector for the cell penetration of a variety of cargoes, including proteins [11], peptides [18], small dyes [16,19], drugs [20,21,22,23] or nanoparticles [24,25]. The mechanism of cell penetration most likely includes a combination of membrane translocation (direct passage to the cytoplasm) and endocytosis, mostly macropinocytosis (indirect access to the cytoplasm through leakage from late endosomes) [16,19,26,27].

Even if the mechanism(s) of penetration of CPP are subject to debate [28,29], because of the rapidity of action of MCa on the ryanodine receptor, it appears clearly that one way of penetration of MCa can be membrane translocation. Furthermore, MCa has the ability to bind onto glycosaminoglycans, including heparin and heparan sulfates, with micromolar affinity. However, cell penetration still occurs according to both mechanisms of penetration in cells devoid of glycosaminoglycans, suggesting that glycosaminoglycans do not preferentially direct MCa’s cell penetration towards endocytosis [26]. In contrast, these cell surface receptors appear to be helpful as peptide sinks for increased peptide delivery into cells. More relevant to cell penetration is the fact that MCa binds to a number of membrane lipids, mostly negatively charged ones [26,27], as observed for other CPP [28]. Binding seems to occur with higher affinity (100 nM) in close agreement with the concentration of MCa required for cell penetration. In addition, MCa analogues that penetrate better than wild-type MCa also exhibit a greater affinity for membrane lipids and *vice versa*. Endocytosis becomes predominant with some large cargoes. Conversely, small cargoes seem to have less interference with a translocation mode of entry of CPP. These observations tend to indicate that endocytosis may become the preferential mode of cell entry of MCa if coupled to bulky cargoes that are expected to increase the duration of residency at the plasma membrane. However, although MCa is an extremely efficient vector for cell penetration of impermeable cargoes, it is of limited usefulness in its native conformation. Indeed, complex disulfide bridging may hamper the attachment of some cargoes. The size of the CPP is greater than those used in the literature. Finally, the native pharmacological activity is generally undesirable for most applications, so like other CPPs before, modification of the native MCa was made in order to ease its use *in vivo* as a new delivery system [30]. Canceling MCa’s pharmacological activity turned out to be quite simple due to the fact that structural requirements involved in binding onto the ryanodine receptor are more stringent than for cell penetration. Several chemical strategies turned out to be successful including point mutations [17], blocking MCa’s folding by preventing disulfide bridge formation [18], and producing a D-diastereomer MCa [16]. The second strategy had the advantage to produce a MCa analogue that was simpler to produce since the oxidation/folding step was no longer necessary. However, the resulting peptide turned out to be slightly less efficient in cell penetration than the folded/oxidized MCa, indicating that the correct positioning in space of the various structural determinants of MCa is important to optimize cell penetration. In addition, the unfolded MCa CPP was still thirty three amino acid residues in length. Quite recently, in an attempt to further delimitate the cell penetrating properties of MCa to smaller sequences, a number of unfolded truncated MCa-derived peptides were synthesized and assessed for cell penetration properties [19]. Surprisingly, all truncated peptides turned out to be more efficient than the unfolded full-length MCa for cell penetration, suggesting that each structured domain within the folded/oxidized MCa may provide a specific contribution to the cell penetration of the wild-type peptide. The shortest peptides were nine residues in length and included both the N-terminal and the C-terminal sequences. One of the peptides, MCa_UF1-9_, stood out as atypical since the net charge of the peptide was 0 and its cell penetration properties differed to some extent from the significantly more basic other MCa-derived truncated peptides. Penetration of this peptide occurred at polarized ends of CHO cells. The peptide also showed greater residency times within the plasma membrane. Its penetration did not rely at all on macropinocytosis for cell entry (at least when coupled to a dye as cargo). Finally, penetration of this peptide occurs at lower extracellular concentrations than the more basic peptides derived from MCa [19]. Altogether the properties of MCa_UF1-9_ seemed interesting enough to warrant a more in-depth investigation of its cell penetration properties. We therefore compare herein the properties of this peptide to well-reputed CPP (Tat, penetratin and poly-R) or analogous peptides derived from other toxins of the calcin family. We investigated the properties of a number of point mutated MCa_UF1-9_ analogues, and more specifically the pH-sensitivity of its penetration in order to design pH-sensitive CPP. The data point to new very powerful CPP with unprecedented efficacies and demonstrate the pH-sensitivities of several of our analogues for cell penetration.

## 2. Results and Discussion

### 2.1. A Peptide Derived from the Hydrophobic Face of MCa Behaves as a Highly Competitive CPP

A schematic representation of MCa illustrates that the amino acid sequence 1 to 9 tops the rest of the peptide (yellow residues) and defines an independent more hydrophobic face (Figure 1A). The opposite side of the peptide is highly basic (blue and pink residues) and defines therefore a basic face. Some of the residues involved in binding onto the ryanodine receptor have been defined in the past [17]. They include important residues such as Arg^23^ and Arg^24^ and define the pharmacophore side (residues in red and in pink). Therefore the pharmacophore and basic regions overlap to some degree.

We compared the efficacy of MCa_UF1-9_ for cell penetration within the glioma rat cell line F98 to other very popular peptides, comprising TAT, penetratin (Pen) and poly-Arg (poly-R). All peptides included an additional C-terminal Cys residue that was labeled with the Cy5 fluorochrome which served the purpose of cargo in this study. Of note, the extra Cys residue in MCa_UF1-9_-C-Cy5 is at its natural position as in the folded/oxidized MCa (Cys^10^) where it is linked to Cys^21^ by a disulfide bridge. In contrast, Cys^3^ of MCa_UF1-9_ was replaced by an isosteric 2-aminobutyric acid (Abu) residue to avoid mislabeling by Cy5 in the middle of the sequence. A series of confocal microscopy images were taken immediately after a 2 h incubation of F98 cells with various concentrations of the four peptides tested (MCa_UF1-9_-C-Cy5, TAT-C-Cy5, Pen-C-Cy5 and poly-R-C-Cy5). As shown, cell penetration of MCa_UF1-9_-C-Cy5 is perceptible at concentrations as low as 100 nM, whereas none of the other peptides showed penetration at this concentration (Figure 1B). Penetration was then dose-dependent for all peptides. It was more marked at 333 nM for MCa_UF1-9_-C-Cy5 and started to show up for Poly-R-C-Cy5, while still absent for TAT-C-Cy5 and Pen-C-Cy5. At 1 µM all peptides showed some degree of penetration, the least efficient peptide being TAT-C-Cy5. At 5 µM, F98 cells incubated with TAT-C-Cy5 showed levels of fluorescence that were more or less comparable to those obtained with MCa_UF1-9_-C-Cy5 at 333 nM. These data qualitatively indicated that MCa_UF1-9_-C-Cy5 behaved better than those three popular CPP as far as F98 cells are concerned. Similar results were obtained with CHO cells indicating that these differences in performances between the four peptides do not depend on the cell type studied (data not shown). With regard to cell distribution of the peptides, we did not notice any obvious differences in distribution suggesting that the peptides may all borrow the same mechanisms of cell penetration. However, a more complete investigation on this issue is needed before one comes to a firm conclusion. To more quantitatively compare the CPP, we investigated fluorescence levels by flow cytometry (Figure 1C). The data confirmed the confocal microscopy analyses showing that MCa_UF1-9_-C-Cy5 behaves more potently than other CPP. The following order of penetration efficiency was observed: MCa_UF1-9_-C-Cy5 >> Poly-R-C-Cy5 = Pen-C-Cy5 > TAT-C-Cy5. At 10 µM, MCa_UF1-9_-C-Cy5 penetrates 5.5-fold better than TAT-C-Cy5, 3.6-fold better than Pen-C-Cy5, and 3.5-fold better than Poly-R-C-Cy5.

### 2.2. Randomly Defined Control Peptides Delimit the Threshold Level of an Acceptable Cell Penetration

Our data also illustrate that the control linker-cargo, a single Cys residue linked to Cy5 (C-Cy5), does not penetrate at all into F98 cells (at concentrations up to 33 µM), demonstrating the peptide specificity of cell entry (Figure 1C and D). Another control was also tested, based on a fragment of charybdotoxin (ChTx), a voltage-gated potassium channel blocker, not known previously for cell penetration aptitude. The peptide encompasses the 12 first amino acids of ChTx. Internal Cys residues were replaced by Abu, an additional Cys residue was added at the C-terminus and the resulting peptide labeled with Cy5 as well to yield ChTx_UF1-12_-C-Cy5. Confocal microscopy images do not show any evidence of cell penetration if F98 cells are incubated 2 h with 3 µM ChTx_UF1-12_-C-Cy5 (Figure 1E). However, if a more sensitive and quantitative approach is taken to examine the levels of fluorescence, it becomes obvious that F98 cells take up a defined amount of ChTx_UF1-12_-C-Cy5. This is unlikely to represent the level of binding of this peptide to some cell-surface potassium channels as this fragment is not known to bind potassium channels. In contrast, it may indicate the propensity of some cell types to internalize peptides that present even low affinity for the plasma membrane. A comparison with our least-performing peptide TAT-C-Cy5 indicates that ChTx_UF1-12_-C-Cy5 is less efficient than TAT-C-Cy5, although significant. At 10 µM, the cell entry of this non-conventional CPP is 1.57-fold less than TAT. These findings may question the relevance of some studies reporting the discovery of “new” CPP or alternatively may suggest that ChTx_UF1-12_ can also be considered as a poorly performing CPP.

### 2.3. Pharmacological Blockade of Endocytosis in F98 Cells Affects Poorly MCa_UF1-9_ Cell Entry

**Figure 2:**
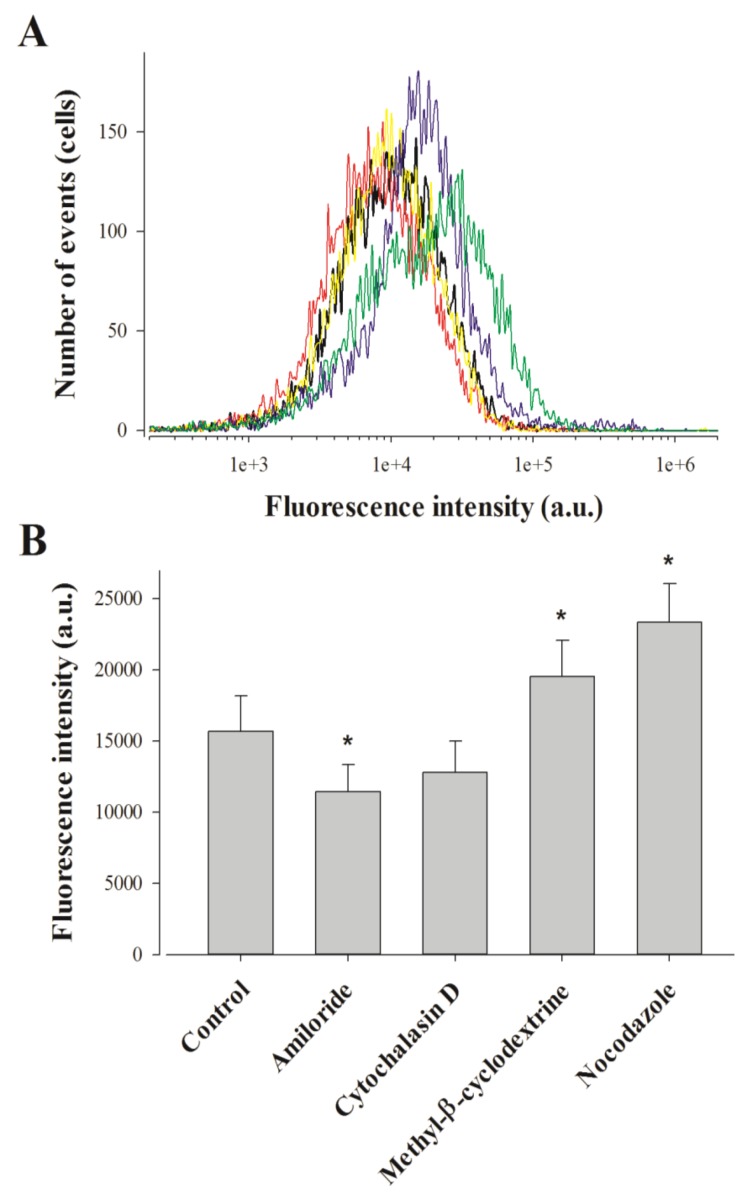
Effect of endocytosis inhibitors on MCa_UF1-9_ peptide penetration in F98 cells. (**A**) Representative FACS data showing the effect of amiloride (red curve), cytochalasine D (yellow curve), methyl-β-cyclodextrine (blue curve) and nocodazole (green curve) on 1 µM MCa_UF1-9_-C-Cy5 cell entry (black curve). (**B**) Average fluorescence intensities for the cell entry of 1 µM MCa_UF1-9_-C-Cy5 in F98 cells without and with endocytosis inhibitors. * *p*≤ 0.1.

Punctiform distribution of MCa_UF1-9_-C-Cy5 may be interpreted as a cell entry that is mainly based on a form of endocytosis. This point was assessed by FACS analyses using various drugs at the 2 h cell entry timepoint of 1 µM MCa_UF1-9_-C-Cy5 in F98 cells. We tested amiloride, a macropinocytosis inhibitor, methyl-β-cyclodextrin to deplete membrane cholesterol and inhibit lipid raft-dependent pathways, nocodazole to prevent microtubule formation, and cytochalasin D to stop F-actin elongation, required for macropinocytosis and clathrin-dependent endocytosis [31]. Amiloride only affected MCa_UF1-9_-C-Cy5 cell entry in F98 cells very mildly, with an average inhibition of 27% indicating an entry partly based on macropinocytosis (Figure 2).

The 18.5% inhibition observed by cytochalasin D, while being non-significant, was in agreement with the contribution of macropinocytosis to the cell entry of the peptide. Quite surprisingly, both methyl-β-cyclodextrin and nocodazole produced 24.7 and 48.9% increases in the cell entry of MCa_UF1-9_-C-Cy5. The lack of inhibition by methyl-β-cyclodextrin indicates that caveolae-mediated endocytosis is not involved in the entry of this peptide. The observed increase in cell penetration may indicate on the contrary that one preferential route of cell entry by translocation may be in lipid rafts. Preventing the loss of lipid rafts by blocking endocytosis at this level would increase the surface area devoted to lipid rafts and hence peptide entry.

Similarly, it is possible that microtubules hinder cell penetration explaining why blocking their formation may help peptide entry to this large extent. These observations demonstrate that while the intracellular distribution of MCa_UF1-9_-C-Cy5 in F98 cells looks punctiform, this is not necessarily the consequence of a cell entry by endocytosis.

### 2.4. Point Mutation of MCa_UF1-9_ Fails to Optimize The Cell Penetrating Properties of This Peptide

**Figure 3:**
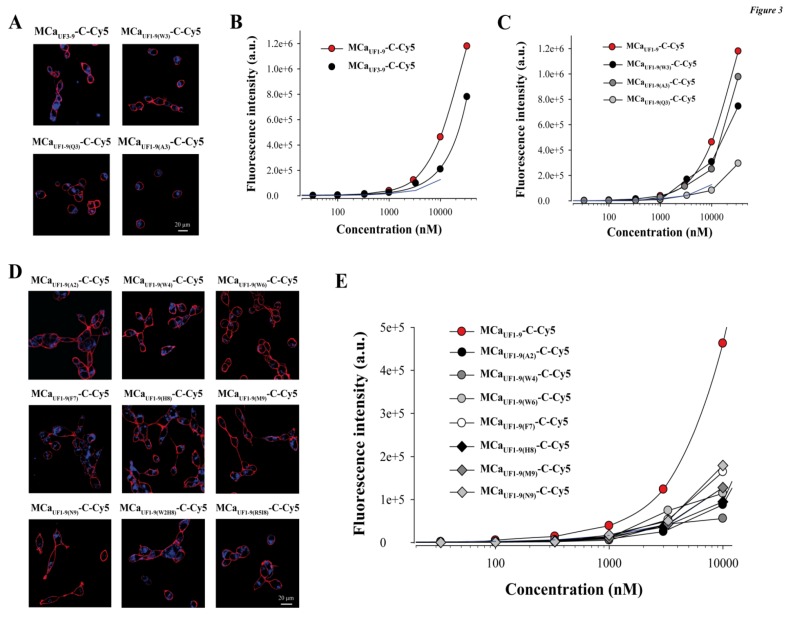
Structural determinants of MCa_UF1-9_ peptide penetration. (**A**) Confocal images illustrating the penetration of 3 µM MCa_UF3-9_-C-Cy5, MCa_UF1-9(W3)_-C-Cy5, MCa_UF1-9(Q3)_-C-Cy5 or MCa_UF1-9(A3)_-C-Cy5 into F98 cells. 2 h incubation time before washout and imaging. (**B**) Effect of N-terminal peptide truncation on cell penetration efficacy of MCa_UF1-9_-C-Cy5 as assessed by flow cytometry. (**C**) Effect of point mutation at position 3 on the cell penetration efficacy of MCa_UF1-9_-C-Cy5. (**D**) Confocal images illustrating the penetration of 3 µM single or double point mutated MCa_UF1-9_-C-Cy5 peptide into F98 cells. (E) Effect of single point mutations on the cell penetration efficacy of MCa_UF1-9_-C-Cy5 as assessed by flow cytometry.

Next, we attempted to design a number of MCa_UF1-9_ peptide analogues in order to get some hints on what structural determinants may be important for the efficacy of this peptide in cell penetration. At the N-terminal side of Cys^3^ (replaced by Abu) of MCa_UF1-9_, there are two residues that appear of minor importance (Gly^1^ and Asp^2^). Removing these two residues yields MCa_UF3-9_. As shown, MCa_UF3-9_-C-Cy5 still accumulates very well in F98 cells (Figure 3A,B).

At the quantitative level, there was a 1.25-decrease in cell penetration efficacy at 3 µM, indicating that these two residues were most likely not essential for cell penetration. This deletion brings the size of this efficient CPP down to seven amino acid residues which is remarkably short. At position 3 of the wild-type MCa there is normally a Cys residue that engages itself into a disulfide bridge. The lateral chain of this residue is therefore not exposed towards the outside face of the molecule and is unlikely to play a role in cell penetration. MCa_UF1-9_ contains an Abu residue instead of a Cys residue and the peptide is quite efficient for cell penetration. We nevertheless probed this position by replacing the Abu residue by Trp, Gln or Ala. As shown, none of these replacements at position 3 within MCa_UF1-9_-C-Cy5 hindered the cell penetration of these analogues (Figure 3A). At 3 µM, MCa_UF1-9(W3)_-C-Cy5 penetrated 1.36-fold better than MCa_UF1-9_-C-Cy5, while MCa_UF1-9(A3)_-C-Cy5 penetrated 1.08-fold less well, indicating little variations (Figure 3C). In contrast, when Gln was put at position 3 in the sequence, the resulting MCa_UF1-9(Q3)_-C-Cy5 peptide behaved similarly to TAT but 2.94-fold less well than MCa_UF1-9_-C-Cy5 suggesting that a Gln may hinder the cell penetration process. Next, we made a series of single (seven peptide) or double (two peptide) point mutated analogues to probe the functional importance of these MCa residues. As shown, all mutated MCa_UF1-9_-C-Cy5 analogues produced evident cell penetration at 3 µM, indicating that none of the substitutions were powerful enough to fully prevent cell penetration (Figure 3D). However, according to flow cytometry analyses, none of the mutated peptides performed better than MCa_UF1-9_-C-Cy5 (Figure 3E). Taking TAT-C-Cy5 as a standard, MCa_UF1-9(A2)_-C-Cy5, MCa_UF1-9(W4)_-C-Cy5 and MCa_UF1-9(H8)_-C-Cy5 behaved slightly less well. In contrast, MCa_UF1-9(F7)_-C-Cy5 and MCa_UF1-9(M9)_-C-Cy5 still behaved better than TAT-C-Cy5. Double mutants (MCa_UF1-9(W2H8)_-C-Cy5 and MCa_UF1-9(R5I8)_-C-Cy5 were also closely similar to TAT-C-Cy5 (not shown). Overall, these data indicate that MCa_UF1-9_ peptide has been optimized for cell penetration with many amino acid residues playing an important role for cell entry. At this stage it would be difficult to point to one single residue as being more important than another one within the sequence.

### 2.5. Analogous Hydrophobic Domains of Other Toxin Members of the Calcin Family Are Also Excellent CPP

**Figure 4:**
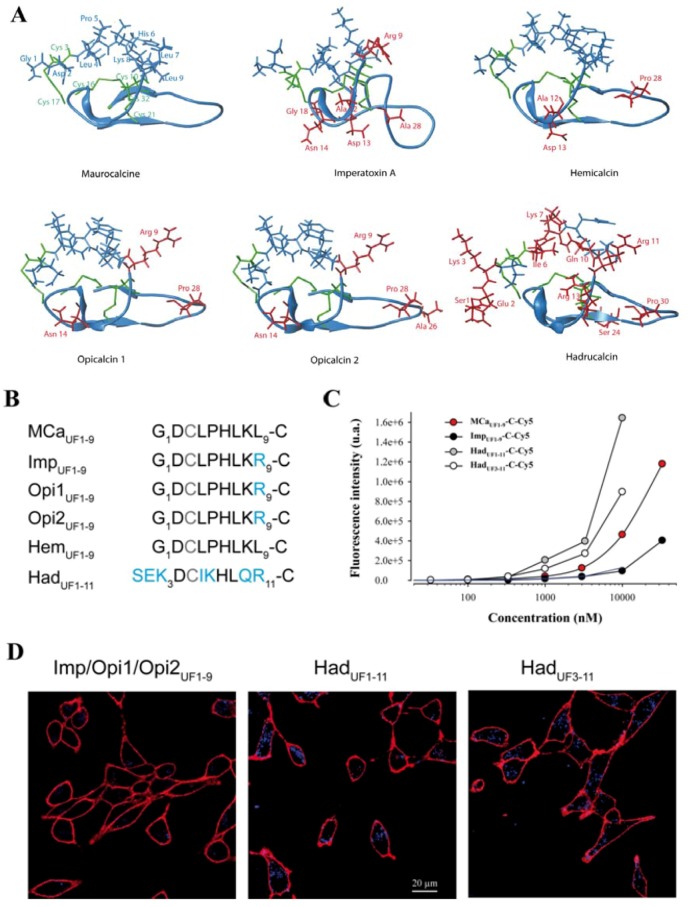
Cell penetration properties of the hydrophobic domains of toxins from the calcin family. (**A**) Real or modeled 3D structures of MCa (PDB access code 1C6W), imperatoxin A (access code 1IE6), hemicalcin (model), opicalcin 1 (model), opicalcin 2 (model) and hadrucalcin (model). Residues in blue describe the hydrophobic domain investigated in this study. Red residues are all amino acids that differ from MCa’s amino acid sequence. Residues in green are cystine residues. (**B**) Amino acid sequences of the hydrophobic domains of each member of the calcin family. The third Cys residue is systematically replaced by Abu in our synthetic peptides and is represented in grey color. Residues in blue are those that differ from MCa_UF1-9_ amino acid sequence. This sequence alignment defines three groups of peptides with similar N-terminal sequences. (**C**) Comparative cell penetration efficacies of the peptides derived from members of the calcin family as assessed by dose-response curves from flow cytometry data. TAT-C-Cy5 data are indicated by a blue dashed line for comparison. (**D**) Confocal images illustrating the cell penetration of each peptide of the calcin family after incubation of 1 µM of the peptides with F98 cells during 2 h. A significantly lower cell penetration is observed for Imp_UF1-9_-C-Cy5 compared to the two hadrucalcin-derived peptides (Had_UF1-11_-C-Cy5 and Had_UF3-11_-C-Cy5).

The inability to produce MCa_UF1-9_ peptides with greater cell penetration efficacies tends to indicate that sequence variation of this hydrophobic domain needs to be considered more cautiously to design optimized peptides. Interestingly, MCa belongs to a larger family of peptides that have, most of them, never been assessed for cell penetration. Results have been presented indicating that imperatoxin A also behaves as a CPP [32]. All these peptides are structured similarly to MCa, with a similar hydrophobic face topping a more basic face (Figure 4A). All peptides that have been tested are also active on the ryanodine receptor [6,7,33], indicating the presence of a similar pharmacophore. A close examination of the amino acid sequence of the hydrophobic domain reveals only minor sequence diversity among these peptides (Figure 4B). The nine first residues of hemicalcin are identical to MCa. Imperatoxin A, opicalcin 1 and opicalcin 2 differ from MCa only by residue 9 (an arginine instead of a leucine residue).

The most differing peptide sequence is the one derived from hadrucalcin with two extra N-terminal residues. Interestingly, the sequence SerGluLys replaces the Gly^1^ residue of MCa, but Asp^2^ of MCa is conserved. Also, four internal substitutions are noticeable. Leu^4^, Pro^5^, Lys^8^ and Leu^9^ of MCa are substituted by Iso, Lys, Gln and Arg, respectively. Finally, Had_UF1-11_ has an additional basic amino acid compared to MCa_UF1-9_. We next evaluated the ability of these peptides to accumulate into F98 cells. At 1 µM, it was obvious, according to confocal imaging, that Imp_UF1-9_-C-Cy5 (equivalent to Opi1_UF1-9_-C-Cy5 or Opi2_UF1-9_-C-Cy5) is less efficient for cell penetration than MCa_UF1-9_-C-Cy5 and Had_UF1-11_-C-Cy5 (Figure 4D). Interestingly, a similar deletion of the two first amino terminal residues of Had_UF1-9_-C-Cy5 yielded a peptide, MCa_UF3-11_-C-Cy5 with excellent penetration capabilities. Evaluation of dose-dependent penetration by flow cytometry demonstrated that Imp_UF1-9_-C-Cy5 penetrates quantitatively in a similar way than TAT-C-Cy5. For the first time, we also show that Had_UF1-11_-C-Cy5 and Had_UF3-11_-C-Cy5 both penetrate better than MCa_UF1-9_-C-Cy5 (Figure 4C).

This indicates that a series of very selective set of amino acid substitutions are required to improve MCa_UF1-9_ cell penetration properties. They also demonstrate that the calcin family can accommodate some variation in cell penetration efficacy for the activation of the ryanodine receptor. Nevertheless, it remains to be investigated whether the additional basic face and pharmacophore region are necessary to improve the characteristics of cell penetration of imperatoxin A, opicalcin 1 or opicalcin 2. In any case, it is obvious that Had_UF1-11_ is a remarkable cell penetration peptide by the extent of its efficacy compared to the popular peptides challenged in Figure 1.

### 2.6. Cell Penetration of MCa_UF1-9_ is pH-sensitive Owing to the Presence of an His Residue in its Amino Acid Sequence

**Figure 5:**
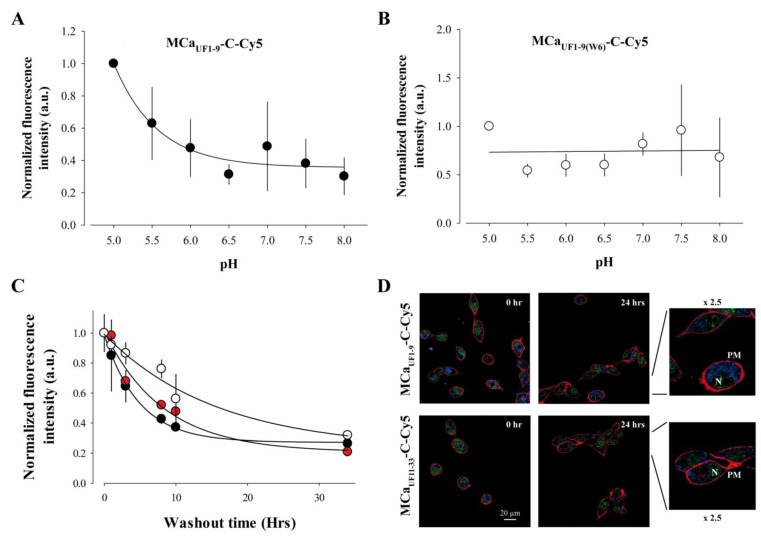
pH-dependence and persistence of the cell penetration of MCa_UF1-9_-C-Cy5. (**A**) Effect of extracellular pH variation on the cell penetration efficacy of 1 µM MCa_UF1-9_-C-Cy5 in F98 cells (2 h incubation). Mean of n = 3 experiments ± S.D. Mean FACS results were normalized before averaging. Data were fitted by a decreasing exponential of the type y = y_0_ + a.e^−bx^ where y_0_ = 0.36 ± 0.05, a = 0.64 ± 0.09 and b = 1.76 ± 0.61. (**B**) Effect of extracellular pH variation on the cell penetration of 1 µM MCa_UF1-9_(W6)-C-Cy5 in F98 cells (2 h incubation). The pH-insensitive Trp^6^ replaces the pH-sensitive His^6^ in this mutant peptide. (**C**) Persistence of the fluorescence signal in F98 cells preincubated 2 h with 1 µM MCa_UF1-9_-C-Cy5 (net charge 0 if His^6^ is unprotonated, black symbol), the positively charged MCa_UF11-33_-C-Cy5 (net charge +7, red symbol) or the mildly charged Had_UF3-11_-C-Cy5 peptide (net charge +2 if His^8^ is unprotonated, white symbol). FACS results were normalized to 1 at t=0 min at the start of the washout of the CPP. Data were fitted with a decreasing exponential function with 1/τ = 0.20 ± 0.01 h^−1^ (MCa_UF1-9_-C-Cy5), 0.12 ± 0.03 h^−1^ (MCa_UF11-33_-C-Cy5) et 0.06 ± 0.02 h^−1^ (Had_UF3-11_-C-Cy5), where τ is the time constant of the decrease in the mean cell fluorescence level. The non-decreasing fraction of fluorescence was equal to 0.27 ± 0.14 (MCa_UF1-9_-C-Cy5), 0.21 ± 0.07 (MCa_UF11-33_-C-Cy5) and 0.23 ± 0.13 (Had_UF3-11_-C-Cy5). (**D**) Corresponding confocal images illustrating that the intracellular distribution of the peptides did not change with time. The 2.5-fold image enhancement also shows a close to complete lack of nucleus invasion by the peptides. The images also show that the two peptides do not differ in the type of intracellular distribution 3.5 h after washout of the extracellular peptides.

Close examination of the amino acid sequence of MCa reveals that it contains a histidine residue at position 6. This residue is therefore also present in MCa_UF1-9_. According to Figure 2 data, this histidine residue contributes to some extent to the cell penetration efficacy of MCa_UF1-9_-C-Cy5. The imidazole sidechain of histidine has a pKa of approximately 6.0, while overall the pKa of the amino acid is 6.5. However, this value is susceptible to be influenced by the direct amino acid environment of this residue. In any case, it can be considered that at physiological conditions, relatively small changes in pH value is susceptible to alter the average charge of MCa_UF1-9_. At a pH value lower than 6, the imidazole ring is essentially protonated. Protonation of the His residue at position 6 may affect the cell penetration efficacy of MCa_UF1-9_-C-Cy5. To test this idea, the extracellular pH value was varied between 5.0 and 8.0 during the 2 h incubation of F98 cells with MCa_UF1-9_-C-Cy5 and the total accumulated fluorescence level evaluated by FACS. The data were normalized to the value at pH 5.0. As shown, decreasing pH values results in higher fluorescence values and therefore greater accumulation of the peptide in F98 cells (Figure 5A).

A fit of the data with a decreasing exponential suggests that the peptide enters into F98 cells with a 2.8-fold lower efficacy than at acidic pH values. Half of this decrease in efficacy occurs for a variation in pH from 5.0 to 5.7 indicating that the pKa value of this histidine residue within MCa_UF1-9_-C-Cy5 may be close to 5.7. The involvement of His^6^ in this pH-dependence of the cell penetration of the peptide is demonstrated by the lack of pH-dependence in cell penetration of the mutant peptide MCa_UF1-9(W6)_-C-Cy5 in which His^6^ is replaced by Trp^6^ (Figure 5B). Overall, these data indicate that protonation of His^6^, provided by acidic environments, results in improved MCa_UF1-9_-C-Cy5 cell delivery. In that respect, it is important to note that F98 cells are from rat glioma and that the extracellular pH within the glioma tumors masses in the brain has been predicted to be acidic [34]. Our finding therefore suggests that the MCa_UF1-9_ peptide may be useful to more specifically deliver anti-tumor drugs within glioma.

### 2.7. Long-Lasting Cell Retention of MCa_UF1-9_

One property of CPP is that they enter quite rapidly into cells. However, the persistence of their intracellular accumulation is seldom looked after. We investigated this question with three peptides. We used MCa_UF1-9_-C-Cy5, a non or poorly charged peptide depending on the protonation level of His^6^, MCa_UF11-33_-C-Cy5, a highly charged peptide mainly encompassing the two C-terminal thirds of MCa, and Had_UF3-11_-C-Cy5 which is less hydrophobic than MCa_UF1-9_-C-Cy5. One could expect that hydrophobic peptides may more readily escape from the cell interior than charged peptides. To test this idea, F98 cells were loaded 2 h with 1 µM of each of these peptides, extensively washed, maintained in culture for a variable duration (between 0 and 34 h, washout time), treated with trypsin and the fluorescence level remaining in the cells estimated by FACS (Figure 5C).

As shown and quite remarkably, the fluorescence levels of the accumulated peptides fade away only slowly with time indicating that the cell entry of the peptides are faster than their cell exit (Figure 5C,D). Time constant of half exit were estimated to be 5 h for MCa_UF1-9_-C-Cy5, 8 h and 20 min for MCa_UF11-33_-C-Cy5 and 16 h and 40 min for Had_UF3-11_-C-Cy5. After 34 h, cells still contained 27, 21 and 23% of MCa_UF1-9_-C-Cy5, MCa_UF11-33_-C-Cy5 and Had_UF3-11_-C-Cy5 fluorescence, respectively. These values are suspected to be higher in fact since 34 h is enough to register at least one cell division cycle. This property of persistence indicates that MCa_UF1-9_, but also MCa_UF11-33_ and Had_UF3-11_, can be used both as CPP and retention agents for drugs that may freely enter into cells but would also freely escape from them. We have previously used such a property to fight against chemo-resistance of the breast tumor cell line MDA-MB-231 by coupling doxorubicin to a non-folded version of MCa [20,22,23]. Confocal microscopy images of the cell distribution of both peptides tested indicate that the distribution of the peptides does not evolve with washout time (Figure 5D). The distribution remains mostly punctiform (with an evolution towards what may seem smaller dots), internal and hardly invades the nucleus.

### 2.8. Discussion

While all MCa truncations and/or disruptions of disulfide bridges produce a loss of the 3D structure, MCa_UF1-9_ is more susceptible than other truncated peptides to preserve some of the structural characteristics that it may possess within the full-length MCa as it encloses by itself the entire hydrophobic face of MCa. At this stage, one may only speculate as to why MCa presents one hydrophobic face diametrically opposite to a highly charged basic face. It may present a strong advantage for the peptide if it has to deal with both a hydrophilic environment (extracellular space and cytoplasm) and a hydrophobic one (membrane lipids). Besides it may be essential to the mechanism of cell translocation if the peptide needs to cope with the amphiphilic nature of membrane lipids. The strong dipole moment of the peptide, resulting from the existence of these two different faces, probably orients the peptide in its interaction with the plasma membrane. The highly basic nature of MCa should also speed up the peptide entry through electrochemical attraction if one considers that it diffuses freely through the plasma membrane, in a similar way that Na^+^ or Ca^2+^ ions would do when permeating through adequate ion channels while attracted by the inside negative membrane potential. Such a mechanism, if proven, would result in peptide accumulation against the concentration gradient. What the effect of a physical separation between these two peptide entities (hydrophobic face and basic face) might be on the cell penetration mechanism remains however unclear. One may notice at this point that MCa_UF1-9_-C-Cy5 and MCa_UF11-33_-C-Cy5 produce quite similar intracellular punctiform distributions in F98 cells arguing that they share nonetheless similar mechanisms of cell penetration. This was also the case for the other CPP investigated in this study (poly-R-C-Cy5, Tat-C-Cy5 and Pen-C-Cy5). It is of interest to note however that the cell distribution of fluorescent D-MCa, a full-length and well-structured analogue of MCa, is mostly diffuse [16] suggesting that combining the hydrophobic and basic faces of the molecule to shorten the residency time in the plasma membrane may represent a significant advantage in cell penetration.

The most interesting information that we could gather from our mutagenesis program of MCa_UF1-9_-C-Cy5 was that the two first amino acid residues were the most dispensable for its penetration properties. This truncation approach led to the design of a 7-mer CPP that has greater potency than most popular CPPs on the market. Investigating the cell penetrating properties of peptides derived from the hydrophobic face of other peptides members of the calcin family turned out as a more interesting approach than mutagenesis. Most of these peptides differ by only a few amino acid residues, with the notable exception of hadrucalcin. Coherent with the mono-substitution we performed at amino acid 9 of MCa_UF1-9_-C-Cy5, the cell penetration properties of Imp_UF1-9_-C-Cy5, Opi1_UF1-9_-C-Cy5 and Opi2_UF1-9_-C-Cy5, all presenting Arg^9^ instead of Leu^9^, were reduced to some extent compared to MCa_UF1-9_-C-Cy5. Remarkably, multiple amino acid substitution as demonstrated within Had_UF1-11_-C-Cy5 resulted in an important and unexpected improvement of cell penetration. Similarly to MCa_UF1-9_-C-Cy5, removing the two first N-terminal amino acid residues of Had_UF1-11_-C-Cy5 produced a peptide with quite significant levels of cell penetration. The core of the sequence, the one we assume to be important for cell penetration (after Abu^3^ in MCa_UF1-9_), contains no less than 4 substitutions (Leu^4^ by Ile, Pro^5^ by Lys, Lys^8^ by Gln, and Leu^9^ by Arg). This indicates that quite elaborate alterations need to be done to MCa_UF1-9_ sequence to further improve it cell penetrating properties. In any case, these findings (i) define a novel CPP with unprecedented efficacy, and (ii) open the door for the design of hadrucalcin/MCa chimeras 1 µM MCa_UF1-9_-C-Cy5 fluorescence at any given concentration and never in terms of starting concentration at which cell entry was observed. These observations suggest that the affinity of these peptides for plasma membrane components remains unaltered.

Since we are interested in developing a number of applications in oncology for MCa analogues, we also investigated the role of His^6^ in cell entry into the glioma F98 cells. Of great interest for future applications, we found that protonation of His^6^, occurring at more acidic pH, but in a range compatible with pH values observed in glioma, produced a three-fold more potent peptide for cell penetration. We assume that protonated His^6^ may form a salt bridge with Asp^2^ in the non-structured MCa_UF1-9_. It is likely that, without this salt bridge, the negative charge carried by Asp^2^ may disfavor cell penetration of MCa_UF1-9_. This observation on the importance of protonation in peptide cell penetration will be useful for the future design of new MCa analogues in which important basic amino acid residues may be substituted by His residues in order to further improve the tumor-selectivity of these potent CPP. Interesting positions will include Lys^19^, Lys^20^, Lys^22^ and Arg^24^ all shown to contribute to the cell penetration efficacy of the full length MCa [17].

## 3. Experimental

### 3.1. Reagents

*N*-α-Fmoc-l-aminoacids, Wang-Tentagel resin and reagents used for peptide syntheses were obtained from Iris Biotech (Marktredwitz, Germany). Solvents were analytical grade products from Acros Organics (Geel, Belgium). Cy5 maleimide mono-reactive dye was purchased from GE Healthcare (Saclay, France).

### 3.2. Peptide Syntheses

Chemical syntheses of truncated toxin peptides were performed as previously described [16]. Briefly, peptides were chemically synthesized by the solid-phase method [35] using an automated peptide synthesizer (CEM^©^ Liberty 12, Matthews, NC, USA). Peptide chains were assembled stepwise on 0.24 meq of Fmoc-D-Arg-Pbf-Wang-Tentagel resin using 0.24 mmol of *N*-α-fluorenylmethyloxycarbonyl (Fmoc) L-amino-acid derivatives. The side-chain protecting groups were: trityl for Cys and Asn, *tert*-butyl for Ser, Thr, Glu and Asp, Pbf for Arg and *tert*-butylcarbonyl for Lys. Reagents were at the following concentrations: Fmoc-amino-acids [0.2 M Fmoc-AA-OH in dimethylformamide (DMF)], activator (0.5 M 2-(1H-benzotriazole-1-yl)-1,1,3,3-tetramethyluronium hexafluorophosphate in DMF), activator base [2 M diisopropylethylamine in N-methylpyrrolidone (NMP)] and deprotecting agent (5% piperazine/0.1 M 1-hydroxybenzotriazole in DMF), as advised by PepDriver (CEM^©^). After peptide chain assembly, resins were treated 4 h at room temperature with a mixture of trifluoroacetic acid/water/triisopropylsilane (TIS)/dithiothreitol (DTT) (92.5/2.5/2.5/2.5). The peptide mixtures were then filtered and the filtrates were precipitated by adding cold *tert*-butylmethyl ether. The crude peptides were pelleted by centrifugation (10,000 × *g*, 15 min) and the supernatants were discarded. Truncated toxin analogues were purified by HPLC using a Vydac C18 column (218TP1010, 25 × 10 cm). Elutions of the peptides were performed with a 10–60% acetonitrile linear gradient containing 0.1% trifluoroacetic acid. The purified fractions were analyzed by analytical RP-HPLC (Vydac C18 column 218TP104, 25 × 4.6 cm). All analogues were characterized by MALDI-TOF mass spectrometry.

### 3.3. Peptide Labeling With Cy5

Each peptide was labeled with Cy5 according to the manufacturer’s protocol (GE Healthcare). Peptides were dissolved at 200 µg/mL in 1 M NaHCO_3_ buffer, pH 9.3. 500 µL of solubilized peptides were added to Cy5-maleimide containing tubes. The mixtures were incubated during 2 h at room temperature and then purified by HPLC using an analytical Vydac C18 column. Elution of the Cy5-labeled peptides was performed with a 5–90% acetonitrile linear gradient containing 0.1% trifluoroacetic acid. The pure peak fractions were lyophilized and peptides quantified by UV spectrophotometer at 650 nm.

### 3.4. Cell Culture

Undifferentiated malignant glioma rat (F98) cell line (from ATCC) was maintained at 37 °C in 5% CO_2_ in DMEM/F-12 nutrient medium (Invitrogen, Cergy Pontoise, France) supplemented with 2% (v/v) heat-inactivated fetal bovine serum (Invitrogen) and 100 µg/mL streptomycine and 100 units/mL penicillin (Invitrogen).

### 3.5. Confocal Microscopy

For analysis of the cell entry of Cy5-labeled-truncated toxin peptides in living cells, cell cultures were incubated with the fluorescent peptides (in DMEM/F-12 nutrient medium only) for 2 h, and then washed twice with phosphate-buffered saline (PBS) alone. The plasma membrane was stained with 50 μg/mL rhodamine-conjugated concanavalin A (Invitrogen, Cergy Pontoise, France) for 5 min. Cells were washed once more. Live cells were then immediately analyzed by confocal laser scanning microscopy using a Zeiss LSM operating system. Rhodamine (561 nm) and Cy5 (633 nm) were sequentially excited and emission fluorescence were collected.

### 3.6. Fluorescence Activated Cell Sorting Analyses

F98 cells were incubated with various concentrations of Cy5-labeled peptides in DMEM/F-12 culture medium without serum at 37 °C for 2 h. The cells were then washed with PBS to remove excess extracellular peptide and treated with 0.48 mM versene (Invitrogen) for 5 min at 37 °C to detach cells from the surface, and centrifuged at 200 *g* in DMEM/F-12 culture medium before suspension in PBS. For experiments concerning endocytosis inhibitors, F98 cells were initially preincubated in DMEM/F-12 culture medium without serum for 30 min at 37 °C with different inhibitors of endocytosis: (i) 100 µM amiloride, (ii) 5 µg/mL cytochalasin D (10 µM), (iii) 20 µM nocodazole, or (iv) 5 mM methyl-β-cyclodextrin (all from Sigma, Lyon, France). The cells were then incubated for 2 h at 37 °C with 1 µM MCa_UF1-9_-C-Cy5 in the presence of each drug. Flow cytometry analyses were performed with live cells using an Accuri C6 flow cytometer (BD Biosciences, Le Pont de Claix, France). Data were obtained and analyzed using CFlow Sampler (BD Biosciences). Live cells were gated by forward/side scattering from a total of 10,000 events.

### 3.7. Molecular Modeling

Using Sybyl X 1.3 (Tripos Inc., St. Louis, MO, USA) and PDB structure of MCa (code 1C6W) and imperatoxin A (code 1IE6), we generated 3D models of opicalcin 1 and 2, hemicalcin and hadrucalcin. There is a sequence homology (76% up to 91%) between these proteins. Based on previous reports [6,7], we replaced some amino acid of MCa sequence to obtain the corresponding ones for the four different proteins. Several steps of minimization and control of the stereochemistry were performed to obtain a model for each molecule.

## 4. Conclusions

In this manuscript, we have demonstrated that MCa_UF1-9_-C-Cy5 starts to show detectable penetration in glioma F98 cells at concentrations as low as 33 nM (5-fold increase over control) as detected by FACS. The process is visible at 100 nM by confocal microscopy and a comparative analysis reveals that it is highly competitive compared to TAT, penetratin or Poly-R CPP. One analogue turns out to be extremely competitive, MCa_UF3-9_, owing to its performance, length and ease of synthesis. Nevertheless, we also demonstrate that engineered optimization of its cell penetrating properties is hard to achieve but that Mother Nature has provided an elegant solution to this problem by selecting itself the best amino acid substitutions under the form of new calcin analogues. In that respect, the hydrophobic domain of hadrucalcin outperforms that of MCa. We evidence for the first time the possibility to modulate peptide cell penetration by external pH provided that His residues are strategically incorporated within the amino acid sequence. This finding enlarges the potential application of these peptides to the treatment of glioma. Additionally, the observation that the residency time of these peptides in glioma F98 cells is quite long suggest that these peptides may be best used when injected once inside a solid tumor rather than by intravenous route.

## References

[1] Fajloun Z., Kharrat R., Chen L., Lecomte C., di Luccio E., Bichet D., El Ayeb M., Rochat H., Allen P.D., Pessah I.N. (2000). Chemical synthesis and characterization of maurocalcine, a scorpion toxin that activates Ca^2+^ release channel/ryanodine receptors. FEBS Lett..

[2] Mouhat S., Jouirou B., Mosbah A., de Waard M., Sabatier J.M. (2004). Diversity of folds in animal toxins acting on ion channels. Biochem J..

[3] Mosbah A., Kharrat R., Fajloun Z., Renisio J.G., Blanc E., Sabatier J.M., El Ayeb M., Darbon H. (2000). A new fold in the scorpion toxin family, associated with an activity on a ryanodine-sensitive calcium channel. Proteins.

[4] Zamudio F.Z., Gurrola G.B., Arevalo C., Sreekumar R., Walker J.W., Valdivia H.H., Possani L.D. (1997). Primary structure and synthesis of imperatoxin A (iptx(a)), a peptide activator of Ca^2+^ release channels/ryanodine receptors. FEBS Lett..

[5] Zhu S., Darbon H., Dyason K., Verdonck F., Tytgat J. (2003). Evolutionary origin of inhibitor cystine knot peptides. FASEB J..

[6] Shahbazzadeh D., Srairi-Abid N., Feng W., Ram N., Borchani L., Ronjat M., Akbari A., Pessah I.N., de Waard M., El Ayeb M. (2007). Hemicalcin, a new toxin from the iranian scorpion hemiscorpius lepturus which is active on ryanodine-sensitive Ca^2+^ channels. Biochem. J..

[7] Schwartz E.F., Capes E.M., Diego-Garcia E., Zamudio F.Z., Fuentes O., Possani L.D., Valdivia H.H. (2009). Characterization of hadrucalcin, a peptide from hadrurus gertschi scorpion venom with pharmacological activity on ryanodine receptors. Br. J. Pharmacol..

[8] Altafaj X., France J., Almassy J., Jona I., Rossi D., Sorrentino V., Mabrouk K., de Waard M., Ronjat M. (2007). Maurocalcine interacts with the cardiac ryanodine receptor without inducing channel modification. Biochem. J..

[9] Szappanos H., Smida-Rezgui S., Cseri J., Simut C., Sabatier J.M., de Waard M., Kovacs L., Csernoch L., Ronjat M. (2005). Differential effects of maurocalcine on Ca^2+^ release events and depolarization-induced Ca^2+^ release in rat skeletal muscle. J. Physiol..

[10] Gurrola G.B., Arevalo C., Sreekumar R., Lokuta A.J., Walker J.W., Valdivia H.H. (1999). Activation of ryanodine receptors by imperatoxin A and a peptide segment of the II-III loop of the dihydropyridine receptor. J. Biol. Chem..

[11] Esteve E., Mabrouk K., Dupuis A., Smida-Rezgui S., Altafaj X., Grunwald D., Platel J.C., Andreotti N., Marty I., Sabatier J.M. (2005). Transduction of the scorpion toxin maurocalcine into cells. Evidence that the toxin crosses the plasma membrane. J. Biol. Chem..

[12] Tanabe T., Beam K.G., Adams B.A., Niidome T., Numa S. (1990). Regions of the skeletal muscle dihydropyridine receptor critical for excitation-contraction coupling. Nature.

[13] Tanabe T., Beam K.G., Powell J.A., Numa S. (1988). Restoration of excitation-contraction coupling and slow calcium current in dysgenic muscle by dihydropyridine receptor complementary DNA. Nature.

[14] Altafaj X., Cheng W., Esteve E., Urbani J., Grunwald D., Sabatier J.M., Coronado R., de Waard M., Ronjat M. (2005). Maurocalcine and domain a of the II-III loop of the dihydropyridine receptor Ca_v_1.1 subunit share common binding sites on the skeletal ryanodine receptor. J. Biol. Chem..

[15] Chen L., Esteve E., Sabatier J.M., Ronjat M., de Waard M., Allen P.D., Pessah I.N. (2003). Maurocalcine and peptide a stabilize distinct subconductance states of ryanodine receptor type 1, revealing a proportional gating mechanism. J. Biol. Chem..

[16] Poillot C., Dridi K., Bichraoui H., Pecher J., Alphonse S., Douzi B., Ronjat M., Darbon H., de Waard M. (2010). D-maurocalcine, a pharmacologically inert efficient cell-penetrating peptide analogue. J. Biol. Chem..

[17] Mabrouk K., Ram N., Boisseau S., Strappazzon F., Rehaim A., Sadoul R., Darbon H., Ronjat M., de Waard M. (2007). Critical amino acid residues of maurocalcine involved in pharmacology, lipid interaction and cell penetration. Biochim. Biophys. Acta.

[18] Ram N., Weiss N., Texier-Nogues I., Aroui S., Andreotti N., Pirollet F., Ronjat M., Sabatier J.M., Darbon H., Jacquemond V. (2008). Design of a disulfide-less, pharmacologically-inert and chemically-competent analog of maurocalcine for the efficient transport of impermeant compounds into cells. J. Biol. Chem..

[19] Poillot C., Bichraoui H., Tisseyre C., Bahemberae E., Andreotti N., Sabatier J.M., Ronjat M., de Waard M. (2012). Small efficient cell-penetrating peptides derived from scorpion toxin maurocalcine. J. Biol. Chem..

[20] Aroui S., Brahim S., de Waard M., Breard J., Kenani A. (2009). Efficient induction of apoptosis by doxorubicin coupled to cell-penetrating peptides compared to unconjugated doxorubicin in the human breast cancer cell line MDA-MB 231. Cancer Lett..

[21] Aroui S., Brahim S., de Waard M., Kenani A. (2010). Cytotoxicity, intracellular distribution and uptake of doxorubicin and doxorubicin coupled to cell-penetrating peptides in different cell lines: A comparative study. Biochem. Biophys. Res. Commun..

[22] Aroui S., Brahim S., Hamelin J., de Waard M., Breard J., Kenani A. (2009). Conjugation of doxorubicin to cell penetrating peptides sensitizes human breast MDA-MB 231 cancer cells to endogenous trail-induced apoptosis. Apoptosis.

[23] Aroui S., Ram N., Appaix F., Ronjat M., Kenani A., Pirollet F., de Waard M. (2009). Maurocalcine as a non-toxic drug carrier overcomes doxorubicin resistance in the cancer cell line MDA-MB 231. Pharm. Res..

[24] Ram N., Texier-Nogues I., Pernet-Gallay K., Poillot C., Ronjat M., Andrieux A., Arnoult C., Daou J., de Waard M. (2011). *In vitro* and *in vivo* cell delivery of quantum dots by the cell penetrating peptide maurocalcine. Int. J. Biomed. Nanosci. Nanotechnol..

[25] Stasiuk G.J., Tamang S., Imbert D., Poillot C., Giardiello M., Tisseyre C., Barbider E.L., Fries P.H., de Waard M., Reiss P., Mazzanti M. (2011). Cell-permeable ln(III) chelate-functionalized InP quantum dots as multimodal imaging agents. ACS Nano.

[26] Ram N., Aroui S., Jaumain E., Bichraoui H., Mabrouk K., Ronjat M., Lortat-Jacob H., de Waard M. (2008). Direct peptide interaction with surface glycosaminoglycans contributes to the cell penetration of maurocalcine. J. Biol. Chem..

[27] Boisseau S., Mabrouk K., Ram N., Garmy N., Collin V., Tadmouri A., Mikati M., Sabatier J.M., Ronjat M., Fantini J. (2006). Cell penetration properties of maurocalcine, a natural venom peptide active on the intracellular ryanodine receptor. Biochim. Biophys. Acta.

[28] Jones A.T., Sayers E.J. (2012). Cell entry of cell penetrating peptides: Tales of tails wagging dogs. J. Control. Release.

[29] Lindgren M., Hallbrink M., Prochiantz A., Langel U. (2000). Cell-penetrating peptides. Trends Pharmacol. Sci..

[30] Lundberg P., Langel U. (2003). A brief introduction to cell-penetrating peptides. J. Mol. Recognit..

[31] Mano M., Teodosio C., Paiva A., Simoes S., Pedroso de Lima M.C. (2005). On the mechanisms of the internalization of s4(13)-pv cell-penetrating peptide. Biochem. J..

[32] Gurrola G.B., Capes E.M., Zamudio F.Z., Possani L.D., Valdivia H.H. (2010). Imperatoxin A, a cell-penetrating peptide from scorpion venom, as a probe of Ca-release channels/ryanodine receptors. Pharmaceuticals (Basel).

[33] El-Hayek R., Lokuta A.J., Arevalo C., Valdivia H.H. (1995). Peptide probe of ryanodine receptor function. Imperatoxin A, a peptide from the venom of the scorpion pandinus imperator, selectively activates skeletal-type ryanodine receptor isoforms. J. Biol. Chem..

[34] Garcia-Martin M.L., Herigault G., Remy C., Farion R., Ballesteros P., Coles J.A., Cerdan S., Ziegler A. (2001). Mapping extracellular pH in rat brain gliomas *in vivo* by 1H magnetic resonance spectroscopic imaging: Comparison with maps of metabolites. Cancer Res..

[35] Merrifield R.B. (1969). Solid-phase peptide synthesis. Adv. Enzymol. Relat. Areas Mol. Biol..

